# Still air resistance during walking and running

**DOI:** 10.1098/rspb.2023.1763

**Published:** 2023-12-13

**Authors:** Matteo M. Pecchiari, Mario A. Legramandi, Giuseppe Gibertini, Giovanni A. Cavagna

**Affiliations:** ^1^ Dipartimento di Fisiopatologia Medico-Chirurgica e dei Trapianti, Università degli Studi di Milano, Milano, Italy; ^2^ Dipartimento di Scienze e Tecnologie Aerospaziali, Politecnico di Milano, Milano, Italy

**Keywords:** air drag, terrestrial locomotion, walking, running

## Abstract

In everyday life during terrestrial locomotion our body interacts with two media opposing the forward movement of the body: the ground and the air. Whereas the work done to overcome the ground reaction force has been extensively studied, the work done to overcome still air resistance has been only indirectly estimated by means of theoretical studies and by measurements of the force exerted on puppets simulating the geometry of the human body. In this study, we directly measured the force exerted by still air resistance on eight male subjects during walking and running on an instrumented treadmill with a belt moving at the same speed of a flow of laminar air facing the subject. Overall, the coefficient of proportionality between drag and velocity squared (*A*_eff_) was smaller during running than walking. During running *A*_eff_ decreased progressively with increasing average velocity up to an apparently constant, velocity independent value, similar to that predicted in the literature using indirect methods. A predictive equation to estimate drag as a function of the speed and the height of the running subject is provided.

## Abbreviations

air density, *ρ*; kinematic viscosity, *ν*; Reynolds number, Re; friction coefficient, *C*_D_; height of the subject, *h*, measured in static conditions; frontal area of the subject, *A*_frontal_; condition in which no wind was present in the wind tunnel, WIND-OFF; condition in which wind was present in the wind tunnel, WIND-ON; centre of mass of the body, COM; acceleration of the centre of mass of the subject in the fore-aft direction, *a*_*f*_; fore-aft ground reaction force measured by the treadmill, *F*_*f*_; vertical ground reaction force measured by the treadmill, *F*_*v*_; lateral ground reaction force measured by the treadmill, *F*_*l*_; velocity of the treadmill or of a subject walking or running outdoor or wind speed ($\bar{V}$: average velocity), *V*; air drag experienced by a subject walking or running outdoor in still air or walking or running on a treadmill against a wind having the same velocity of the treadmill, *F*_drag_; difference between the fore-aft ground reaction force measured by the treadmill and the product of the mass times the acceleration of the center of mass of the subject in the fore-aft direction, extra *F*_*f*_; frictional force related to the displacement of the COM during an eventual viscoelastic deformation of the body, *F*_fr_; force exerted by the subject to accelerate and decelerate the belt, *F*_belt_; effective area, that is the ratio between measured air drag and the product between air density and the average velocity of the wind (as in our experimental conditions average velocity of the wind and of the treadmill were equal), *A*_eff_; effective area that could hypothetically be measured at infinite running speed (see the text for explanations), $A_{\mathrm{eff},\bar{V}\to \infty }$; difference between A_eff_ and $A_{\mathrm{eff},\bar{V}\to \infty }$ that could hypothetically be measured at zero running speed (see the text for explanations), $A_{\mathrm{eff},\mathrm{low}\bar{V}}$; inverse of the speed squared at which $A_{\mathrm{eff},\mathrm{running}}=A_{\mathrm{eff},\bar{V}\to \infty }+0.37A_{\mathrm{eff},\mathrm{low}\bar{V}}$ (see the text for explanations), *b*; proportionality constant between A_eff_ and height squared, *k*_eff_; computational fluid dynamics studies, CFD.

## 1. Introduction

In everyday life, when we walk or run in still air, air exerts a force (a drag) which tends to reduce the velocity of the body in the forward direction and should be counterbalanced by muscle activity in order to maintain the velocity of progression. This force contributes to limit the maximal speed in world-class athletes [1], and accounts for the importance of drafting, a running technique by which an athlete takes advantage of the low pressure wake created by another athlete running in front of him/her [2–5].

Still air resistance has multiple determinants, including air density, the apparent dimensions and the velocity of the subject (i.e. the velocity of the air relative to the subject). Estimation of the drag is difficult, as the human body is a complex structure whose frontal area is constantly changing during locomotion due to the movements of the limbs and of the torso. For these reasons, computational fluid dynamics studies (CFD) [2–6] have indirectly estimated drag representing runners as mannequins frozen in a single running pose. Similarly, experimental studies have measured drag on puppets, boards, equivalent cylinders or humans in static conditions only [7–11], so at present no direct measurement of the drag in walking and running humans is available.

The purpose of the present study is to assess directly the force exerted by still air on a subject walking or running at constant speed. This can be done with a treadmill allowing us to measure the ground reaction force in the fore–aft direction inside a wind tunnel producing a tightly controlled laminar air speed equal to treadmill speed. Indeed, the drag exerted by still air on a subject walking or running on open ground is equal to that exerted on a subject walking or running on a treadmill by a wind blowing with the same velocity and direction as the treadmill but with the opposite orientation. In this condition, since the velocity of the laminar flow of air equals the velocity of the treadmill, the force exerted on the body by air would be nil if the subject were standing immobile on the moving treadmill. On the contrary, if the subject is walking or running, drag can be computed as the difference in average ground reaction force in the fore–aft direction recorded in the absence and in the presence of the wind.

## 2. Methods

Experiments were performed in the low turbulence test section of the Wind Tunnel Laboratory (GVPM, Politecnico di Milano, Italy) (4 m wide × 3.84 m high × 6 m long, turbulence level less than 0.1%). Briefly, a commercial treadmill with belt surface 1.7 × 0.65 m (h/p/Cosmos, Germany) was mounted in the centre of the wind tunnel, so that its longer axis was parallel to the direction of the wind. The motor of the treadmill, bulging above the treadmill surface, was placed behind the subject walking or running against wind, in order not to disturb the laminarity of air flow. The connections between the treadmill and the ground were provided by strain-gauge force transducers (Arsalis, Belgium), which allowed measurement of fore–aft (*F_f_*), vertical (*F_v_*) and lateral (*F_l_*) ground reaction forces [12]. The treadmill was shielded with a wooden framework to reduce the direct action of the wind on the force transducers. For safety reasons, the subject was connected to a loose rope hanging from a pulley on the roof of the tunnel. The additional drag provided by the rope at the maximal wind velocity used (5 m s^−1^) was negligible (< 0.1 N).

Eight male professional runners (age 31 ± 6 years, height 1.77 ± 0.05 m, mass 66 ± 7 kg) participated in this project. Subjects had naked limbs, and a face mask together with a K5 (COSMED, Rome, Italy) fixed on their backs used for other measurements. Each subject walked or ran on the treadmill without wind (WIND-OFF) or against a wind with the same velocity of the treadmill (WIND-ON). Nominal treadmill speeds were 1, 1.5 and 2 m s^−1^ for walking, and 3, 3.5, 4, 4.5 and 5 m s^−1^ for running. One subject was tested at 1.5, 4.0 and 4.5 m s^−1^ only. Before each recording, the outputs of force transducers were zeroed with the treadmill running and the subject standing outside of the treadmill; in WIND-ON sessions, zeroing was performed in the presence of wind with the same velocity (V) as the treadmill. All signals were acquired at 1000 Hz for 60 s in each condition (in some few instances for 30 s), and were analysed with custom-built LabView programs (National Instruments, USA).

The study was approved by the ethics committee of Politecnico of Milan (no. 16/2022), and each participant signed an informed consent to the experiments.

### (a) Analysis

The procedure adopted to measure the drag (*F*_drag_) relies on the fact that instantaneous fore–aft ground reaction force (*F_f_*_(*t*)_), measured by the treadmill, should counterbalance the mass of the body times the acceleration in the fore–aft direction (*a_f_*_(*t*)_) of the centre of mass (COM), and all the other forces applied to the COM in the fore–aft direction (extra*F_f_*_(*t*)_), including frictional forces related to the displacement of the COM during an eventual viscoelastic deformation of the body (*F*_fr(*t*)_) [13], the force exerted by the subject to accelerate and decelerate the belt (*F*_belt(*t*)_) [14] and the drag (*F*_drag(*t*)_). When average velocity is constant, as in a case of a subject walking or running on a treadmill, mean COM acceleration is zero, so that *F_f_*_(*t*)_ averaged in a sufficiently large number of complete steps ${\bar{F}}_{f}={\bar{F}}_{\mathrm{fr}}+{\bar{F}}_{\mathrm{belt}}+{\bar{F}}_{\mathrm{drag}}$. Therefore ${\bar{F}}_{\mathrm{drag}}$ can be obtained by subtracting ${\bar{F}}_{f}$ measured in a WIND-OFF session from ${\bar{F}}_{f}$ measured in the corresponding WIND-ON session, as long as *F*_fr_ and *F*_belt_ are the same in the two conditions.

Practically, ${\bar{F}}_{f}$ was measured by averaging *F_f_*_(*t*)_ in all the steps of a recording after the exclusion of the first 1–2 and of the last 1–2 steps.

### (b) Statistics

Analyses were performed using SPSS 28 (SPSS Inc., Chicago, IL), Sigmaplot 12.5 (Systat Software Inc., San Jose, CA).

Comparisons among experimental conditions were performed using analysis of variance for repeated measurements (a wind factor with two levels (WIND-OFF and WIND-ON), and a velocity factor with eight levels, each corresponding to a velocity).

Relationships between variables were assessed by means of linear or nonlinear regression analysis.

Results are given as mean ± SD (standard deviation) except for equation coefficients, which are given as coefficient ± SE (standard error). The level for statistical significance was taken at *p* < 0.05.

### (c) Technical considerations

The measurement of still air resistance using a force platform depends on some assumptions.

First, biomechanics of locomotion should be similar indoor on the treadmill and outdoor in still air. This is overall true. Indeed, a recent systematic review has shown that spatio-temporal, kinetic, kinematic, muscle activity and muscle–tendon parameters are very close in the two conditions [15].

Second, wind speed should be tightly coupled with treadmill speed. In our experimental condition the two parameters were very similar, as wind speed was only 2.6 ± 1.6% greater than treadmill speed. A good match between wind and treadmill speed should also reduce the vertical distance from the ground above which local wind velocity becomes equal to the velocity in the centre of the wind tunnel, that is the thickness of the boundary layer. The thickness of the boundary layer is zero in still air, but is different from zero when wind flows on a stationary flat surface [16]. In addition, turbulence should be reduced as much as possible. In our experimental conditions the level of turbulence was less than 0.1%.

Third, all forces applied to the COM in the fore–-aft direction, with the exception of the drag, should be the same in WIND-OFF and WIND-ON sessions. In the present experiments, these forces were not directly measured, however, given the constancy of treadmill speed, step duration and length in WIND-OFF and WIND-ON conditions, it is very unlikely that anelastic deformation of the body or the force exerted by the subject to accelerate and decelerate the belt were different.

Fourth, the wind should not directly affect the forces measured by the treadmill, as this would change ${\bar{F}}_{f}$ measured in the WIND-ON condition, precluding any meaningful estimation of the drag. This eventuality was at least in part prevented by shielding the treadmill with a wooden framework. More importantly, the output of the force transducers was carefully zeroed before each recording in WIND-OFF and WIND-ON conditions, so that the output only reflected ground reaction forces produced by the subject walking or running on the treadmill.

## 3. Results

During experiments, in the wind tunnel barometric pressure was 738 ± 2 mmHg and temperature 28 ± 1°C, corresponding to an air density of 1.131 ± 0.006 kg m^−3^.

Measured treadmill speed was less than the imposed nominal speed, the difference between the two being small at all walking or running speeds (−1.0 ± 0.3 and −1.5 ± 0.4% of imposed nominal velocity, respectively). Belt speed variations were not greater than 6% of the average speed. In WIND-ON sessions, wind velocity was greater than treadmill velocity by a trivial amount (0.07 ± 0.06 m s^−1^, 2.6 ± 1.6%).

No difference was detected between WIND-OFF and WIND-ON sessions in treadmill velocity (*P*_wind factor_ = 0.249, *P*_interaction_ = 0.419). Similarly, step duration and length were the same (*P*_wind factor_ = 0.555, *P*_interaction_ = 0.287 and *P*_wind factor_ = 0.306, *P*_interaction_ = 0.274, respectively) in the two conditions.

**Figure 1.**
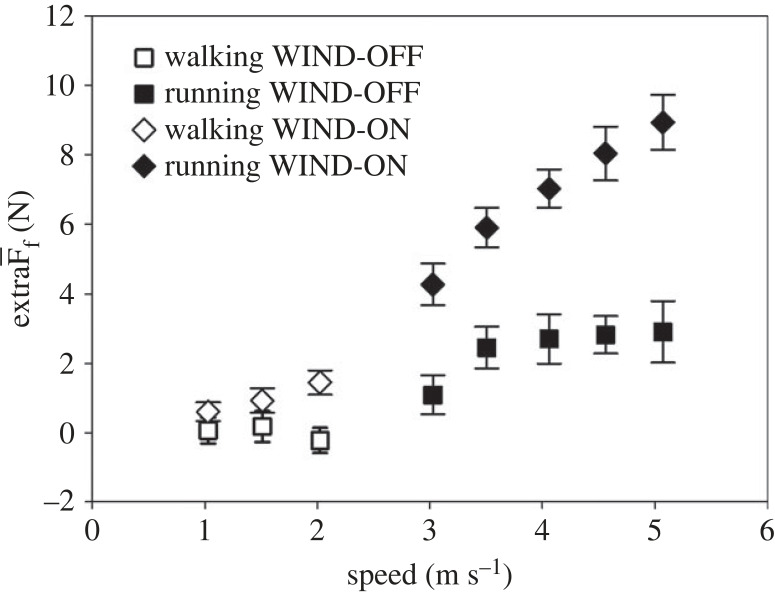
${\bar{F}}_{f}$ during walking (open symbols) and running (closed symbols) as a function of speed without (squares) and with wind (diamonds) in eight subjects. Bars are s.d. ${\bar{F}}_{f}$ was greater than zero with the exception of walking without wind.

During WIND-OFF, ${\bar{F}}_{f}$ was not significantly different from zero at all walking velocities (minimum *p* > 0.153), but increased at all running velocities (*p* < 0.002) (figure 1). On the contrary, in the WIND-ON condition, ${\bar{F}}_{f}$ was significantly greater than zero at all speeds (*p* < 0.001) and greater than ${\bar{F}}_{f}$ at corresponding speed without wind, the difference increasing with increasing speed (*P*_wind factor_ < 0.001, *P*_interaction_ < 0.001) (figure 1).

**Figure 2.**
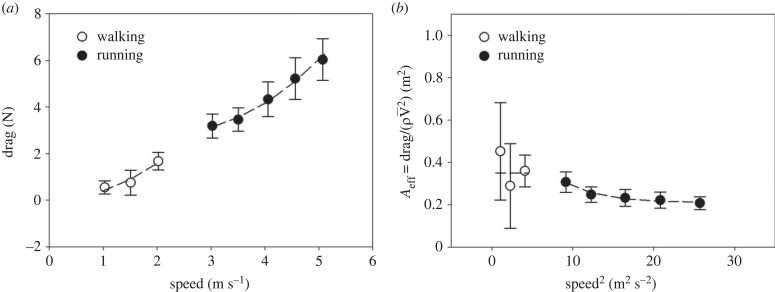
(*a*) Relation between air drag during walking (open circles) and running (closed circles) and average speed. The broken lines are ${\bar{F}}_{\mathrm{drag}}=\rho \;0.349\;{\bar{V}}_{}^{2}$ for walking and ${\bar{F}}_{\mathrm{drag}}=\rho \;(0.211+0.801\;\;e^{-0.236{\bar{V}}^{2}}){\bar{V}}^{2}$ for running. (*b*) Relation between effective area (A_eff_) and average speed squared, that is the ratio between air drag and the product of air density (*ρ*) and average velocity squared (${\bar{V}}_{}^{2}$). The broken lines are *A*_eff_ = 0.349 for walking and $A_{\mathrm{eff}}=0.211+0.801\;\;e^{-0.236{\bar{V}}^{2}}$ for running.

The difference between ${\bar{F}}_{f}$ measured during WIND-ON and WIND-OFF, namely the drag due to the air, is shown in figure 2*a*.

On the ground of the known proportionality between drag and velocity squared [2–5,7–10], the relation between speed and drag during walking and running has been characterized by calculating effective area (*A*_eff_) as

3.1 $$A_{\mathrm{eff}}=\frac{{\bar{F}}_{\mathrm{drag}}}{\rho \;\;{\bar{V}}_{}^{2}},$$

where $\bar{V}$ is average speed, and *ρ* is the air density (*A*_eff_ would be equivalent to one half of product C_D_*A*_frontal_, if both drag coefficient (C_D_) and frontal area (*A*_frontal_) were invariant during a step).

The results are shown in figure 2*b*.

Overall, *A*_eff_ was smaller during running than walking (*p* = 0.003).

If the relation between drag and average speed squared were linear, as previously suggested [7,8], *A*_eff_ would be constant for both walking and running. For walking, this may be possible, as no significant difference between A_eff_ at different walking speeds can be detected (*p* = 0.392). Assuming velocity-independence of A_eff_ during walking, experimental data can be described by the following equation:

3.2 $${\bar{F}}_{\mathrm{drag},\mathrm{walking}}=\rho \;A_{\mathrm{eff},\mathrm{walking}}\;{\bar{V}}_{}^{2}$$

where *A*_eff,walking_ is 0.349 ± 0.028 m^2^ (R^2^ = 0.600)

On the other hand, during running *A*_eff_ decreases progressively with increasing average velocity up to an apparently constant, velocity independent, value (*p* = 0.043), the difference between *A*_eff_ at the slowest and at the highest average velocity being significant (*p* = 0.002).

On this ground, the relationship between *A*_eff_ and average velocity squared during running has been tentatively described with a three parameters exponential decay of the form:

3.3 $$A_{\mathrm{eff},\mathrm{running}}=A_{\mathrm{eff},\bar{V}\to \infty }+A_{\mathrm{eff},\mathrm{low}\bar{V}}\;\;e^{-b{\bar{V}}^{2}},$$

where $A_{\mathrm{eff},\bar{V}\to \infty }$ is the *A*_eff_ that could be measured at infinite speed, $A_{\mathrm{eff},\mathrm{low}\bar{V}}=A_{\mathrm{eff},\mathrm{running}}-A_{\mathrm{eff},\bar{V}\to \infty }$ at V = 0 m s^−1^, and b is the inverse of the speed squared at which $A_{\mathrm{eff},\mathrm{running}}=$ $A_{\mathrm{eff},\bar{V}\to \infty }+1/2.718\;A_{\mathrm{eff},\mathrm{low}\bar{V}}=A_{\mathrm{eff},\bar{V}\to \infty }+0.37A_{\mathrm{eff},\mathrm{low}\bar{V}}$. Nonlinear regression using equation (3.3) was performed on the data shown in figure 2*b* during running. The resulting coefficients were $A_{\mathrm{eff},\bar{V}\to \infty }=0.211\pm 0.0145\;m^{2}$, $A_{\mathrm{eff},\mathrm{low}\bar{V}}=0.801\pm$ $0.909\;m^{2}$, and *b* = 0.236 ± 0.130 s^2^ m^−2^ (*R*^2^ = 0.446) (broken line in figure 2*b*).

According to previous work, in humans drag increases linearly with increasing height squared (h^2^) of the subject, as this parameter is an estimator of the frontal area [7,17].

**Figure 3.**
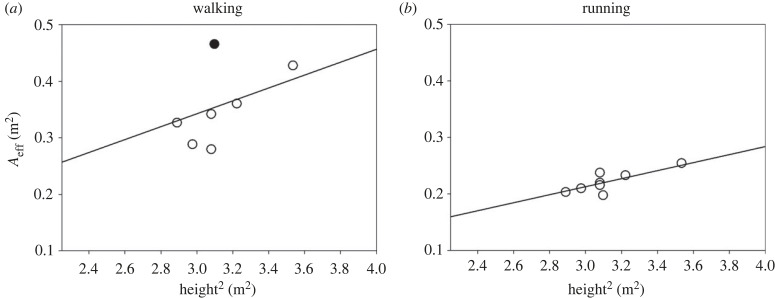
Relation between A_eff_ and height squared in (*a*) seven walking and (*b*) eight running subjects (open circles). In one subject, A_eff_ could not be calculated during walking as only one walking speed was recorded. A possible outlier in (*a*) is indicated with a filled circle.

The relation of *A*_eff_ and h^2^ in our walking subjects is shown in figure 3*a*. This relation was not significant (*P*_slope_ = 0.196, *P*_intercept_ = 0.592, *R*^2^ = 0.309), possibly because of the presence of one outlier, indicated as a filled circle (the residual of this data point was greater than two times the standard deviation of all residuals). If the outlier is not considered, the slope of the *h*^2^–*A*_eff_ becomes significant (*P*_slope_ = 0.037), while the intercept remains not different from zero (*P*_intercept_ = 0.232, *R*^2^ = 0.703).

In order to be able to compare our results with the predictions from CFD and wind-tunnels studies, the outlier has been removed, and a relation of the form *A*_eff_ = *k*_eff,walking_ *h*^2^ assumed, where *k*_eff,walking_ is a proportionality constant. Substituting *A*_eff_ in equation (3.1) with *k*_eff,walking_ h^2^, we have that:

3.4 $${\bar{F}}_{\mathrm{drag},\mathrm{walking}}=\rho \;k_{\mathrm{eff},\mathrm{walking}}\;h^{2}\;{\bar{V}}_{}^{2},$$

where *k*_eff,walking_ is 0.108 ± 0.011.

A similar procedure has been made to assess the dependency of *A*_eff_ on *h*^2^ during running. In this case, however, *A*_eff_ has been calculated in each subject at the three highest speeds, as in this speed range the velocity dependence of *A*_eff_ is small (figure 2*b*). Despite the limited range of the heights of the participants (1.70–1.88 m), individual *A*_eff_ values were linearly related to the square of the height during running (*P*_slope_ = 0.015, *P*_intercept_ = 0.715, R^2^ = 0.656) (figure 3*b*).

Considering the dependency of *A*_eff_ on *h*^2^ during running, multiple nonlinear regression was performed with *A*_eff_ as the dependent variable, and ${\bar{V}}^{2}$ and *h*^2^ as the predictors, using an equation of the form of equation (3.3), where $A_{\mathrm{eff},\bar{V}\to \infty }$ was substituted by $k_{\mathrm{eff},\bar{V}\to \infty }h^{2}$ and $A_{\mathrm{eff},\mathrm{low}\bar{V}}$ by $k_{\mathrm{eff},\mathrm{low}\bar{V}}h^{2}$:

3.5 $$A_{\mathrm{eff},\mathrm{running}}=h^{2}(k_{\mathrm{eff},\bar{V}\to \infty }+k_{\mathrm{eff},\mathrm{low}\bar{V}}\;\;e^{-b{\bar{V}}^{2}}).$$

According to this analysis, $k_{\mathrm{eff},\bar{V}\to \infty }=0.067\pm 0.005$, $k_{\mathrm{eff},\mathrm{low}\bar{V}}=0.216\pm 0.219$, and *b* = 0.218 ± 0.118 s^2^ m^−2^ (*R*^2^ = 0.502). Consequently, still air resistance during running can be expressed as

3.6 $${\bar{F}}_{\mathrm{drag},\mathrm{running}}=\rho \;h^{2}(k_{\mathrm{eff},\bar{V}\to \infty }+k_{\mathrm{eff},\mathrm{low}\bar{V}}\;\;e^{-b{\bar{V}}^{2}}){\bar{V}}^{2}.$$

**Figure 4.**
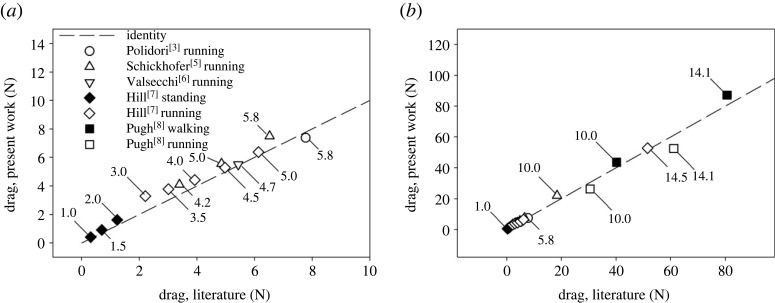
Relation between the drag calculated by CFD [3,5,6] or measured in a wind tunnel [7,8] in static conditions and that predicted in dynamic conditions according to equations (3.4) and (3.6). (*a*) Comparisons referring to velocities similar to those used in the present experiments. (*b*) Comparisons at velocities much higher than those used in the present experiments. Broken lines are identity lines. The numbers indicated for each data point are wind speeds.

Equations (3.4) and (3.6) have been used to compare the expected drag present in dynamic conditions with static estimates of the drag in previous studies based on CFD or measurements in a wind tunnel [3,5–8], using the height of the models and the densities of air provided by the authors. The results are shown in figure 4.

## 4. Discussion

In this work we present the first direct measurement of the drag experienced by a subject walking or running in still air. Previously, this parameter has been estimated in static conditions only, either in a wind tunnel [7–10], or by CFD [2–6,16].

The direct measurement of still air drag allowed us to calculate *A*_eff_, that is the proportionality constant between average drag and average speed squared at each velocity. The relation between drag and speed is more commonly expressed in terms of friction coefficient (*C*_D_) and frontal area (*A*_frontal_) as

4.1 $$F_{\mathrm{drag}}=\frac{1}{2}C_{D}A_{\mathrm{frontal}}\rho \;V^{2},$$

where 1/2*ρV*^2^ is the dynamic pressure. In the real world all these parameters, with exception of density, are expected to change instant by instant during walking or running, because of changes of instantaneous speed, body shape and frontal area during a step, even if average velocity is constant. How the instantaneous changes of C_D_, *A*_frontal_ and *V* affect average drag during walking and running is, to our knowledge, unknown. Pugh estimated that the changes of *A*_frontal_ during running are relatively small, amounting to approximately 6% [18], and Crouch *et al.* [19] found minor differences in terms of drag between a pedaling cyclist and a stationary one. These considerations suggest that instantaneous drag can be not so different from average drag, but, at present, this remains a speculation.

According to our results, *A*_eff_ is on the average greater during walking than running. A similar conclusion was reached by a previous experimental study [8], and by a recent CFD study [20]. It is tempting to explain this result based on greater frontal area during walking than during running [7,8], but likely this is only a partial explanation, as Hill noted that a difference in frontal area could not completely explain the different drags produced by the wind on his puppet in standing and running postures [7].

The scatter of the drags measured during walking appears much larger than during running, as graphically shown by the error bars in figure 2. A straightforward explanation for this finding is that measurement error was larger in the former than in the latter condition. Indeed, drag is just a tiny fraction of fore–aft ground reaction forces generated during locomotion. Moreover, with increasing speed, drag increases faster than peak *F*_f(t)_ (at our maximal running speed drag is approximately 1.6% of peak *F*_f(t)_, at our lowest walking speed the same percentage is less than 0.5%). In this situation, any disturbance of the recorded signal is likely to produce a error in measured drag greater during walking than during running. It is not however possible to exclude that at least part of the scatter during walking is related to changes of C_D_ or *A*_frontal_ at the different velocities. Equation (3.4) has been formulated assuming constant *A*_eff_, but this has been done for data description and comparison purposes, and we recognize that more experiments should be performed for a complete characterization of *A*_eff_ as a function of velocity during walking.

During running data scatter appears markedly reduced, and the solidity of our estimates of the drag is suggested by the significant relation between *A*_eff_ and the height of the subjects, despite the limited range of heights available. Figure 2*b* shows that overall A_eff_ decreases with increasing average speed up to an apparently constant value. This seems in contrast with previous findings suggesting a linear relation during running between drag and speed squared [7,8].

On one hand, this discrepancy can be simply due to the fact that drag has been previously assessed on models at relatively high wind velocities only, in a range in which *A*_eff_ is constant and the drag-speed squared relation is almost a straight line (figure 2*b*). On the other hand, this phenomenon can be related to limb motion, present in our experiments but absent in the previous one.

In its general appearance this trend can be explained as a Reynolds (Re) number dependency. At lower speeds a large part of the flow is not completely turbulent, and the Reynolds number defined on a macro-scale (in this case it could be the subject's height so that Re=Vh/ν, *ν* being kinematic viscosity) is a good measure of the inverse of viscous stresses relative importance. Indeed, both for their direct effect resulting in a friction force and for the influence on the position of the flow separations, the viscous stresses are strongly related to the drag. Thus a general trend of drag area decreasing as the speed increases is expectable. On the other hand, when the Reynolds number becomes so large that the flow is fully turbulent, energy dissipation mainly occurs at the turbulent microscale level and tends to an asymptotic behaviour.

To the best of our knowledge, no previous work reporting air resistance during walking and running in dynamic conditions, namely in the presence of limb movement relative to the COM, can be found in the literature. Comparisons are possible only with the estimates from CFD and models obtained in static conditions, as shown in figure 4*a* (for wind velocities similar to those used in present experiments) and figure 4*b* (for greater wind velocities). The results obtained by Hill with a puppet in the standing position [7] have been included in the comparison, on the assumption that the drag existing in this position is similar to that during walking. Overall, drag assessed in static conditions in the literature, although of the same order of magnitude, tends to underestimate that measured in the present study in dynamic conditions (Valsecchi *et al*., [6], Pugh, [8] during walking, Hill, [7], Schickhofer & Hanson, [5]). By contrast, Pugh overestimated drag during running by approximately 16% [8]. Regarding these comparisons, a major limitation to be kept in mind is that we estimated the drag that should have been present in the conditions of previous studies using the reported height of the subjects according to equations (3.4) and (3.6). Although height is related to the aerodynamic dimensions of the subjects [7,17], variations in the dimensions of the subjects at a given height have surely an effect on the drag, reducing the predictive ability of equations (3.4) and (3.6). In our professional runners body mass alone did not outperform height squared as a predictor of *A*_eff_ during running (*R*^2^ = 0.415, *p* = 0.085). When weight and height squared together were used in the form of body surface area [21], no improvement of *A*_eff_ prediction was detected (*R*^2^ = 0.501, *p* = 0.049). If any, height squared appears the best predictor of *A*_eff_ during running at high speeds, but caution should be applied due to the limited number of subjects, and the small range of BMIs considered (18–22 kg m^−2^). Indeed, an empirical equation based on body mass reported in [1] predicts with a less than 7% error the average drag measured in our subjects during running at high speeds.

**Figure 5.**
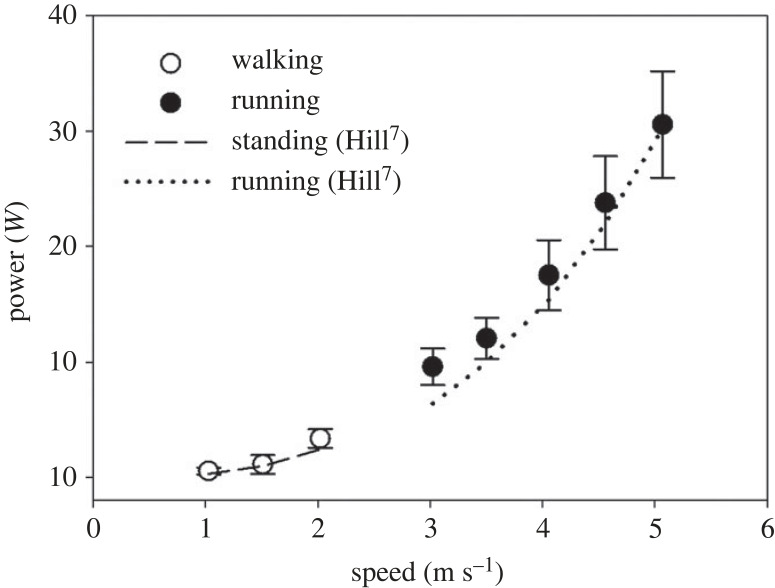
Relation between speed and the power that should be produced to overcome air resistance, calculated as drag times speed in the present subjects during walking (open circles) and running (closed circles), and multiplying speed times the drag predicted by Hill for standing and running (broken and dotted lines, respectively).

Given the remarkable similarity between our estimates of the drag and those of Hill at high running speed, it is not surprising that also the estimates of the power, which should be produced to overcome air resistance, are very close (figure 5).

## Data Availability

The data on which this study is based are provided in the electronic supplementary material [22].
